# Accelerated hyperfractionated intensity-modulated radiotherapy for recurrent/unresectable rectal cancer in patients with previous pelvic irradiation: results of a phase II study

**DOI:** 10.1186/s13014-014-0278-3

**Published:** 2014-12-11

**Authors:** Gang Cai, Ji Zhu, Weigang Hu, Zhen Zhang

**Affiliations:** Department of Radiation Oncology, Fudan University Shanghai Cancer Center, Shanghai, 200032 China

**Keywords:** Rectal cancer, Local recurrence, Reirradiation, Hyperfractionation, Intensity-modulated radiotherapy

## Abstract

**Background:**

This study was conducted to investigate the local effects and toxicity of accelerated hyperfractionated intensity-modulated radiotherapy for recurrent/unresectable rectal cancer in patients with previous pelvic irradiation.

**Methods:**

Twenty-two patients with recurrent/unresectable rectal cancer who previously received pelvic irradiation were enrolled in our single-center trial between January 2007 and August 2012. Reirradiation was scheduled for up to 39 Gy in 30 fractions using intensity-modulated radiotherapy plans. The dose was delivered via a hyperfractionation schedule of 1.3 Gy twice daily. Patient follow-up was performed by clinical examination, CT/MRI, or PET/CT every 3 months for the first 2 years and every 6 months thereafter. Tumor response was evaluated 1 month after reirradiation by CT/MRI based on the RECIST criteria. Adverse events were assessed using the National Cancer Institute (NCI) common toxicity criteria (version 3.0).

**Results:**

The median time from the end of the initial radiation therapy to reirradiation was 30 months (range, 18-93 months). Overall local responses were observed in 9 patients (40.9%). None of the patients achieved a complete response (CR), and 9 patients (40.9%) had a partial response (PR). Thirteen patients failed to achieve a clinical response: 12 (54.5%) presented with stable disease (SD) and 1 (4.5%) with progressive disease (PD). Among all the patients who underwent reirradiation, partial or complete symptomatic relief was achieved in 6 patients (27.3%) and 13 patients (59.1%), respectively. Grade 4 acute toxicity and treatment-related deaths were not observed. The following grade 3 acute toxicities were observed: diarrhea (2 patients, 9.1%), cystitis (1 patient, 4.5%), dermatitis (1 patient, 4.5%), and intestinal obstruction (1 patient, 4.5%). Late toxicity was infrequent. Chronic severe diarrhea, small bowel obstruction, and dysuria were observed in 2 (9.1%), 1 (4.5%) and 2 (9.1%) of the patients, respectively.

**Conclusions:**

This study showed that accelerated hyperfractionated intensity-modulated radiotherapy significantly relieved local symptoms and led to a promising local response with an acceptable toxicity profile in patients with recurrent/unresectable rectal cancer and previous pelvic irradiation. Innovative treatment regimens should be evaluated in future studies to improve the clinical outcome while avoiding excessive toxicity in patients with recurrent rectal cancer and previous pelvic irradiation.

## Introduction

Despite recent advances in pretreatment radiological evaluation, total mesorectal excision, radiotherapy, and chemotherapy, 5-10% of patients with primary rectal cancer develop locally recurrent rectal cancer (LRRC) [1-4]. LRRC can cause severe symptoms, including pelvic pain, bleeding, and bowel obstruction, which significantly impact the quality of life, and patient prognosis remains poor [5,6]. Salvage therapy for LRRC remains a challenge. Although radical surgery is the only approach with a curative intent, only approximately 20-30% of patients with recurrent rectal cancer can undergo an R0 resection [7]. Most patients cannot undergo curative surgery because of locally unresectable disease, lack of medical fitness, an unwillingness to accept the high postoperative morbidity and mortality rates [7], or the presence of unresectable extrapelvic metastases.

Radiation is an effective approach for relieving symptoms and improving local control in patients with recurrent rectal cancer. However, some patients with recurrent rectal cancer have previously received radiation, which increases the complexity and difficulty of the management. Reirradiation is often not recommended because of the extremely high incidence of complications in normal tissues. Although data regarding reirradiation outcomes are scarce, several recent observations and trials have demonstrated the safety and efficacy of reirradiation in patients with recurrent rectal cancer who previously underwent irradiation [8-13]. To reduce the potential risk of late toxicity in normal tissues related to previous radiation, hyperfractionated radiation may be a good option. In addition, NCCN guidelines recommend that intensity-modulated radiotherapy (IMRT) be used in the context of reirradiating patients with recurrent disease [14]. However, to the best of our knowledge, no prospective studies have evaluated the efficacy and safety of accelerated hyperfractionated IMRT in patients with recurrent rectal cancer and previous pelvic irradiation. Based on these considerations, we performed a phase II study to investigate the local effects and safety of accelerated hyperfractionated IMRT in patients with recurrent rectal cancer who previously received pelvic irradiation.

## Materials and methods

### Patients

Twenty-two patients with recurrent/unresectable rectal cancer and previous pelvic irradiation were enrolled between January 2007 and August 2012 in our single-center trial. This prospective study was approved by our institutional review board (Fudan University Shanghai Cancer Center), and all the patients signed informed consent forms.

To be eligible, patients had to present with histologically confirmed primary rectal adenocarcinoma. Each patient was discussed, evaluated, and determined to have unresectable cancer by our multidisciplinary team before reirradiation, and a pelvic recurrence was diagnosed by histological confirmation or based on the typical appearance on PET/computed tomography (CT), CT, or MRI. All the patients had received previous pelvic irradiation. There were measurable lesions in the fields of radiotherapy. The other inclusion criteria were as follows: age between 18 and 75 years; Eastern Cooperative Oncology Group performance score ≤2; adequate hematological and liver function; leucocytes >4.0 × 10^9^/L; platelets >100 × 10^9^/L; bilirubin <1.5× the upper limit of normal (ULN); aspartate aminotransferase/alanine aminotransferase ≤2.5× ULN; and serum creatinine <1.25× ULN.

The exclusion criteria were as follows: any other malignancy; significant coronary or cardiac conditions; serious uncontrolled infection; a psychiatric disorder; and pregnancy or lack of contraceptive use in women with child-bearing potential.

### Pretreatment evaluation

The pretreatment workup was performed within the two weeks prior to initiating the reirradiation and included a complete history, physical examination, digital rectal examination, colonoscopy (if possible), tumor biopsy (if possible), chest CT, abdominal CT, pelvic CT or MRI or PET/CT, and complete laboratory tests.

### Radiotherapy

Radiotherapy was delivered with a linear accelerator using 6-MV photons. Each patient had a planning CT scan in the treatment position (prone position using a belly board or supine position). IMRT planning was performed for all the patients based on the planning CT. Target definition followed the recommendations of ICRU report No. 83 [15]. The target volumes and the nearby at-risk organs were delineated on the Pinnacle 8.0 planning system. The gross tumor volume (GTV) was determined based on a combination of the findings from the physical exam, CT, MRI, and/or PET/CT. The planning target volume (PTV) was generated with a 2-3 cm margin around the GTV to avoid as much of the bladder and small bowel as possible. The treatment plans were optimized such that more than 98% of the PTV was completely encompassed by the 93% isodose line while maintaining a minimum dose greater than 90% and a maximum dose less than 110%. The small bowel, bladder, and femoral heads were defined as at-risk organs. The tolerances of normal tissues were defined as follows: 1) Small bowel: No more than 180 cc above 20 Gy, no more than 65 cc above 30 Gy, and maximum dose less than 40 Gy; 2) Femoral heads: No more than 40% volume above 25 Gy, no more than 25% volume above 30 Gy, and maximum dose less than 40 Gy; and 3) Bladder: No more than 40% volume above 25 Gy, no more than 15% volume above 30 Gy, and maximum dose less than 40 Gy. Inverse planning with five coplanar IMRT fields was constructed. The dose was delivered twice daily, 5 days per week, with a hyperfractionation schedule of 1.3 Gy and an interval of at least 6 h between fractions. Thirty fractions to 39 Gy were prescribed.

### Treatment after reirradiation

After reirradiation, subsequent treatment was recommended as necessary but was not included in the study protocol. Subsequent treatment (chemotherapy or surgery) was individualized with no specific recommendations.

### Study design and data evaluation

The primary endpoints of this study were local response and symptom relief after accelerated hyperfractionated IMRT for recurrent rectal cancer in patients with previous pelvic irradiation. Secondary endpoints included toxicity and follow-up.

The patients were routinely followed with clinical examinations, CT/MRI, or PET/CT every 3 months for the first 2 years and every 6 months thereafter. Tumor responses were evaluated 1 month after reirradiation by CT/MRI according to the RECIST criteria. Adverse events were assessed using the National Cancer Institute (NCI) common toxicity criteria (version 3.0).

The different parameters for each individual patient were entered into a database and were analyzed using SPSS 17.0 statistical software. Local progression-free survival rates and overall survival rates were determined using the Kaplan-Meier method.

## Results

### Patient characteristics

Twenty-two patients with recurrent rectal cancer who previously received pelvic irradiation were enrolled between January 2007 and August 2012 in our single-center trial. The study population had a median age of 53 years (range, 40-68 years). Nine participants were men (40.9%), and 13 (59.1%) were women.

Table 1 summarizes the patient characteristics at primary diagnosis and their prior treatment. The initial staging was as follows: 3 stage I patients, 2 stage II patients, and 17 stage III patients. A total of 11 patients (50.0%) underwent low anterior resection, 10 (45.5%) underwent abdominoperineal resection, and 1 (4.5%) underwent local excision. In addition, 17 patients received postoperative chemotherapy consisting of capecitabine or 5-FU plus oxaliplatin. Previous irradiation consisted of preoperative irradiation in 3 patients, postoperative irradiation in 11 patients, and irradiation at first recurrence in 8 patients. The previous radiation doses to the pelvis ranged from 36 to 62 Gy, with a median of 48.6 Gy.

**Table 1:** **Patient characteristics at primary diagnosis and previous treatment (n = 22)**

**Characteristics**	**No.**	**%**
Gender		
Male	9	40.9
Female	13	59.1
T stage at primary diagnosis		
T1	4	18.2
T2	1	4.5
T3	12	54.5
T4	5	22.7
Primary N stage		
N0	5	22.7
N1	9	40.9
N2	8	36.4
Primary stage		
I	3	13.6
II	2	9.1
III	17	77.3
Initial surgery		
Low anterior resection	11	50.0
Abdominoperineal resection	10	45.5
Local excision	1	4.5
Previous radiation		
Preoperative	3	13.6
Postoperative	11	50.0
Recurrent	8	36.4
Previous radiation dose		
Median (Gy)	48.6	-
Range (Gy)	36-62	-

The patient characteristics at reirradiation are presented in Table 2. The interval between the initial surgery and the first pelvic recurrence ranged from 13 to 100 months, with a median of 29 months. The median time from the end of the initial radiation to reirradiation was 30 months (range, 18-93 months). Four patients with recurrent rectal cancer had simultaneous extrapelvic metastases at the time of reirradiation, whereas 18 patients did not present with extrapelvic metastasis. Thirteen patients underwent reirradiation at the first recurrence, and 9 patients underwent reirradiation at the second recurrence. There were a total of twenty-five recurrent sites among all the patients: 20 patients had a single site, and 2 patients had multiple sites. With respect to location, 5 sites were in the perirectal region, 7 in the presacral region, 7 in the internal iliac nodal region, 5 in the perineum, and 1 in the external iliac nodal region. After reirradiation, 18 patients received 5-FU based chemotherapy, and none of these patients underwent a subsequent surgical resection.

**Table 2:** **Patient characteristics at reirradiation (n = 22)**

**Characteristics**	**No.**	**%**
Age		
Median (y)	53	-
Range (y)	40-68	-
Recurrent sites		
Single	20	90.9
Multiple	2	9.1
Recurrence location (n = 25*)		
Perirectal region	5	20.0
Presacral region	7	28.0
Internal iliac nodal region	7	28.0
Perineum	5	20.0
External iliac nodal region	1	4.0
Frequency of recurrence		
First recurrence	13	59.1
Second recurrence	9	40.9
Reirradiation interval		
Median (months)	30	-
Range (months)	18-93	-
≤2 year	7	31.8
>2 year	15	68.2
Size of largest recurrence		
≤3 cm	5	22.7
>3 cm	17	77.3
CEA level		
Normal (<5 ng/mL)	4	18.2
Increased (≥5 ng/mL)	18	81.8
Extra pelvic metastases		
No	18	81.8
Yes	4	18.2
Chemotherapy after reirradiation		
No	4**	18.2
Yes	18	81.8

*Multiple locoregional recurrences present in 2 patients (the perirectal region and the perineum in one patient; the presacral region, internal iliac nodal region, and perineum in another patient).

**Subsequent chemotherapy was recommended for all patients, but 4 patients did not undergo chemotherapy due to intestinal obstruction (2 patients), progressive disease during reirradiation (1), and refusal (1).

### Local response and clinical symptom relief

All the patients with recurrent rectal cancer who previously underwent pelvic irradiation were evaluated for local responses 1 month after reirradiation using CT/MRI. In total, 9 patients (40.9%) achieved a partial response (PR); there was no complete response. Thirteen patients failed to achieve a clinical response, with 12 (54.5%) presenting with stable disease (SD), and 1 (4.5%) presenting with progressive disease (PD) (Table 3).

**Table 3:** **Local response rates and clinical symptom relief**

**Characteristics**	**No.**	**%**
Local response		
Complete response (CR)	0	0
Partial response (PR)	9	40.9
Stable disease (SD)	12	54.5
Progressive disease (PD)	1	4.5
Symptom relief*		
Complete relief	6	27.3
Partial relief	13	59.1
No relief	3	13.6

*All of the patients had severe clinical symptoms before reirradiation, including pain (17 patients), bleeding (1 patient), mass effect (1 patient), pain and bleeding (2 patients), and bleeding and mass effect (1 patient).

All the patients had severe clinical symptoms before reirradiation, including pain (17 patients), bleeding (1 patient), mass effect (1 patient), pain and bleeding (2 patients), and bleeding and mass effect (1 patient). Complete or partial clinical symptom relief after reirradiation was achieved in 6 patients (27.3%) and 13 patients (59.1%), respectively. Clinical symptom palliation was achieved for a median duration of 10 months (range, 3-20 months) (Table 3).

### Toxicity and dose intensity

Table 4 shows the incidence of acute toxicity during reirradiation. Grade 4 toxicity and treatment-related deaths were not observed. Diarrhea was the most common side effect. Grade 3 toxicities included diarrhea in 2 patients (9.1%), cystitis in 1 patient (4.5%), radiation dermatitis in 1 patient (4.5%), and an intestinal obstruction in 1 patient (4.5%).

**Table 4:** **Acute toxicity**

**Toxicity**	**Grade 1 no. (%)**	**Grade 2 no. (%)**	**Grade 3 no. (%)**	**Grade 4 no. (%)**
Diarrhea	3 (13.6)	4 (18.2)	2 (9.1)	0 (0)
Cystitis	2 (9.1)	4 (18.2)	1 (4.5)	0 (0)
Radiation dermatitis	0 (0)	1 (4.5)	1 (4.5)	0 (0)
Intestinal obstruction	0 (0)	0 (0)	1 (4.5)	0 (0)

A total of 20 patients (90.9%) received the planned dose (39 Gy) of accelerated hyperfractionated reirradiation as scheduled (mean relative dose intensity, 95.8%). The reirradiation was terminated for 2 patients: one developed a grade 3 intestinal obstruction, and the other experienced progressive disease. The one patient with a grade 3 intestinal obstruction achieved symptom resolution after adopting conservative treatment. Six patients (27.3%) required a break in the reirradiation course, with a median delay of 2 days (range, 1-6 days), due to acute toxicity.

The incidence of late toxicity was infrequent. Only 2 patients developed severe chronic diarrhea. One patient developed a small bowel obstruction and was hospitalized for occasional tube decompression. Two patients developed dysuria and required percutaneous nephrostomy drainage. None of the patients developed skin ulceration.

### Disease control and survival

The median follow-up for all the patients after completing the reirradiation was 17 months (range, 2-59 months). At the time of the last follow-up, clinical local control had been achieved in 7 patients (31.8%), and 13 patients had developed distant metastases. The cumulative local progression-free survival rates were 67.0% (SE, ±10.3%) at 1 year post-reirradiation and 10.7% (SE, ±9.2%) at 2 years post-reirradiation. The median local control time was 14 months. Eighteen patients (81.8%) died, and the cumulative overall survival rates were 85.9% (SE, ±7.6%) at 1 year post-reirradiation and 27.2% (SE, ±10.3%) at 2 years post-reirradiation. The median survival time after reirradiation was 19 months.

## Discussion

The published literature offers limited data on the use of reirradiation for treating recurrent rectal cancer in patients with previous pelvic irradiation. To the best of our knowledge, no previous prospective studies have evaluated the efficacy and safety of accelerated hyperfractionated IMRT in recurrent rectal cancer patients with previous pelvic irradiation. This study determined whether accelerated hyperfractionated IMRT is effective and safe for treating recurrent/unresectable rectal cancer in patients with previous pelvic irradiation. The overall local response rate was 40.9%, and the clinical symptom relief rate was 86.4%. Grade 4 acute toxicity and treatment-related deaths were not observed. Grade 3 diarrhea, cystitis, radiation dermatitis, and intestinal obstruction were observed in 9.1%, 4.5%, 4.5%, and 4.5% of the participants, respectively. Late toxicity was infrequent and mild.

Reirradiation for recurrent rectal cancer has historically been unacceptable for patients with previous pelvic irradiation because of the extremely high incidence of complications in normal tissues. Data on reirradiation are scarce. However, several recent reports and trials have demonstrated the safety and efficacy of reirradiation for treating recurrent rectal cancer in patients with previous pelvic irradiation [8,10-13]. Although a high reirradiation dose cannot be applied to previously irradiated regions, reirradiation doses of 30-40 Gy are well tolerated and provide decent palliation in most patients. Recently, hyperfractionated reirradiation has been used for recurrent rectal cancer to reduce the risk for late toxicity in normal tissues related to previous irradiation. Several studies have suggested that hyperfractionated reirradiation is a valuable treatment option that provides effective palliation with an acceptable toxicity profile [11,12]. Additionally, to reduce toxicity in normal tissues, IMRT has been recommended when reirradiating patients with recurrent rectal cancer [14]. Several dosimetric and clinical studies have shown that IMRT for rectal cancer can reduce the dose delivered to the small bowel (thereby reducing treatment-related toxicity) compared with standard 3DCRT [16,17]. Based on these considerations, accelerated hyperfractionated IMRT was used to treat patients with recurrent rectal cancer after previous pelvic irradiation.

Although reirradiation concurrent with chemotherapy may lead to better clinical outcomes, we did not evaluate this regimen in our study. Mohiuddin et al. [10] reported high toxicity in patients concurrently treated with reirradiation and continuous 5-FU infusion. In their study, 28% of the patients developed grade 3 to 4 acute toxicity during reirradiation, and 21% of the patients developed late complications, such as chronic severe diarrhea and small bowel obstruction. This acute toxicity required interrupting or terminating the treatment in 22% of the patients. In our study, the total reirradiation course was only 3 weeks, and the impact of concurrent chemotherapy may have been small. Moreover, in our study, the intent of reirradiating patients with inoperable recurrent rectal cancer was palliative.

Our study achieved an overall local response rate of 40.9%. None of the patients achieved a CR, mainly due to the large tumor sizes (77.3% of the patients had lesions larger than 3 cm) and the low total reirradiation dose. These findings are comparable to those reported in previous trials. Valentini et al. [11] reported results from a multicenter phase II study of reirradiation in 59 patients with recurrent rectal cancer. These patients were treated twice daily with 1.2-Gy fractions for a cumulative dose of 40.8 Gy and with concurrent continuous 5-FU infusion. The authors reported a 44.1% overall response rate (CR, 8.5%; PR, 35.6%). Another retrospective study [13] investigated reirradiation in 22 patients with recurrent rectal cancer who had previously received pelvic irradiation. The overall response rates at 1 and 3 months were 32% and 41%, respectively. A CR was observed in one patient (5%) at 3 months. The percentage of PRs increased from 27% at 1 month to 37% at 3 months.

Reirradiation provided effective symptomatic palliation, even at the lower total dose that was administered to the recurrent rectal tumors. In our study, the clinical symptom relief rate was 86.4% (complete symptom relief rate, 27.3%; partial symptom relief rate, 59.1%). The proportion of patients achieving clinical symptomatic relief was similar to that reported in several other reirradiation studies. Mohiuddin et al. [10] reported that bleeding, pain, and the effects of the tumor were positively impacted in 100%, 82.6%, and 88.9% of the patients, respectively, after reirradiation. Valentini et al. [11] also reported a high symptom relief rate (83.3%) 4 weeks after the end of the reirradiation and concurrent chemotherapy.

In our study, the toxicity data revealed that accelerated hyperfractionated IMRT was well tolerated, with low rates of acute and late toxicity, in patients with recurrent rectal cancer and previous pelvic irradiation. Diarrhea was the most common acute toxicity; late toxicity was infrequent and mild. Various acute and late toxicities have been described in the literature, and most studies have reported a low incidence of toxicity, similar to our results [11,13]. However, Mohiuddin et al. [10] reported that 22% of the patients required a significant treatment interruption, and 15% of the patients terminated treatment due to severe acute toxicities. Additionally, grade 4 diarrhea was observed in 6% of the patients, and late complications were reported for 21.4% of the patients. Das et al. [12] also reported a high incidence of grade 3 to 4 late toxicity (26%). The incidence of reirradiation toxicity is correlated with many factors, such as reirradiation dose, reirradiation fractionation, total cumulative dose, the amount of small bowel that is exposed to the radiation field, reirradiation intervals, and the previous radiation and reirradiation volumes. When reirradiating the pelvis to treat recurrent rectal cancer, every effort should be made to limit the dose to the small bowel and bladder in the radiation field.

Although reirradiation of recurrent rectal cancer can result in excellent palliation of symptoms and good local responses with acceptable toxicity, the long-term local control and survival remain poor. To improve these factors, other approaches, such as radiotherapy dose escalation, radiotherapy combined with newer chemotherapeutic and biological agents, intraoperative radiotherapy, hyperthermia, radical surgery, and systemic treatment, should be investigated in future studies.

Our study had several limitations. One limitation is that the sample size was small. Furthermore, late toxicity may have been incompletely assessed based on medical records. Moreover, some patients who might have developed late toxicity died prematurely. Therefore, the late toxicity rates may have been underestimated.

In conclusion, accelerated hyperfractionated IMRT can provide excellent symptom palliation and a good local response in patients with recurrent/unresectable rectal cancer and previous pelvic irradiation. The toxicity profiles for the dosage and schedule used in our study were acceptable. Additional studies are necessary to develop innovative treatment regimens and improve the clinical outcome while avoiding excessive toxicity in patients with recurrent rectal cancer who received previous pelvic irradiation.
